# Toward noninvasive optoacoustic imaging of whole-heart dynamics in mice

**DOI:** 10.1038/s41377-025-01992-x

**Published:** 2025-11-27

**Authors:** Sandeep Kumar Kalva, Cagla Özsoy, Daniil Nozdriukhin, Savannah Tiemann, Lin Tang, Xosé Luís Deán-Ben, Daniel Razansky

**Affiliations:** 1 Institute of Pharmacology and Toxicology and Institute for Biomedical Engineering, Faculty of Medicine, University of Zurich, Zurich, Switzerland; 2 Institute for Biomedical Engineering, Department of Information Technology and Electrical Engineering, ETH Zurich, Zurich, Switzerland; 3 Department of Biosciences and Bioengineering, Indian Institute of Technology Bombay, Mumbai, 400076 India

**Keywords:** Photoacoustics, Imaging and sensing

## Abstract

High-speed volumetric optoacoustic tomography (VOT) offers powerful means for noninvasive, detailed visualization of rapid cardiac dynamics in mice. However, current implementations suffer from non-uniform light delivery into the thoracic area, which results in diminished penetration depth, limited field-of-view, and compromised quantification abilities. In this work, we devised a new VOT approach featuring hexagonally-shaped light delivery optimized for whole-heart imaging and an expedited imaging speed of 200 volumes per second using a custom-made spherical array transducer. The enhanced imaging performance was confirmed with calibration phantoms and noninvasive imaging of the murine heart. We capitalized on the reduced hemoglobin absorption in the second near-infrared (NIR-II) spectral window to mitigate the strong light attenuation by whole blood within the cardiac chambers while further employing copper sulfide nanoparticles featuring a strong NIR-II absorption to quantify cardiac functional parameters across the entire heart in vivo. The new approach can thus facilitate the monitoring of cardiac abnormalities and assessment of therapeutic interventions.

## Introduction

Cardiovascular disease remains a leading cause of mortality worldwide, emphasizing the continuous need for a better understanding and diagnosing heart abnormalities at early stages. To this end, non-invasive preclinical cardiac imaging has been performed with multiple modalities offering complementary advantages. The common imaging performance parameters of particular importance for cardiac applications include spatio-temporal resolution, imaging depth, field-of-view (FOV), as well as the imaging contrast mechanism. For instance, optical (fluorescence) imaging can measure electrical activity through transmembrane potential and intracellular calcium in isolated (Langendroff) perfused heart models^1,2^ whereas pulse-echo ultrasound (US) can complement fluorescence readings by additionally visualizing mechanical wave propagation^3^. US is also a mainstay modality for in vivo cardiac imaging, mainly owing to its superb temporal resolution unattainable with other full-body imaging modalities such as magnetic resonance imaging (MRI) and computed tomography (CT) that commonly rely on gated acquisitions to resolve heart dynamics and assess blood flow^4,5^. Recently, optoacoustic tomographic imaging methods have been proposed as a promising approach for cardiovascular imaging in mice capitalizing on the synergistic advantages of optical (absorption-based) contrast, excellent spatio-temporal resolution, and centimeter-scale penetration depth unattainable with other optical approaches^6^.

A unique advantage of the optoacoustic method is its capability of generating volumetric (3D) images with a single laser pulse, which enables reaching an unprecedented temporal resolution theoretically only limited by the US time-of-flight and practically constrained by the per-pulse energy and pulse repetition frequency of the excitation laser. High-frame-rate imaging has been used for assessing blood perfusion dynamics based on extrinsically administered contrast agents^7–9^. In vivo volumetric optoacoustic tomography (VOT) of the heart has been achieved with spherical transducer arrays by operating in the so-called first near-infrared window (NIR-I, 650–1000 nm), where light penetration is enhanced with respect to the visible light spectrum. The availability of dyes featuring optical absorption within the NIR range was further exploited to visualize perfusion through the heart chambers and quantify the pulmonary transit time (PTT), defined as the duration it takes for blood to travel from the right-sided to the left-sided circulation^10–13^. However, the strong light absorption by the whole-blood concentrated in the heart chambers hampers whole-heart VOT imaging in vivo. Reaching deeper locations can be facilitated by operating in the second near-infrared window (NIR-II, 1000–1400 nm) spectrum, where hemoglobin absorption and light scattering in biological tissues is further reduced^14,15^. The suboptimal illumination strategies employed in the current VOT systems further limit the achievable FOV, imaging depth, and signal quantification capabilities^16^. These shortcomings can partially be mitigated by mechanical scanning of the spherical array^17–19^, which naturally comes to the detriment of the achievable temporal resolution.

In this work, we developed a new VOT approach featuring hexagonally-shaped light delivery optimized for whole-heart imaging via uniform illumination of the chest area. We further assessed the performance of contrast-enhanced VOT imaging with polyethylene glycol (PEG)-coated copper sulfide (CuS) nanoparticles featuring strong absorption in the NIR-II.

## Results

**Fig. 1:**
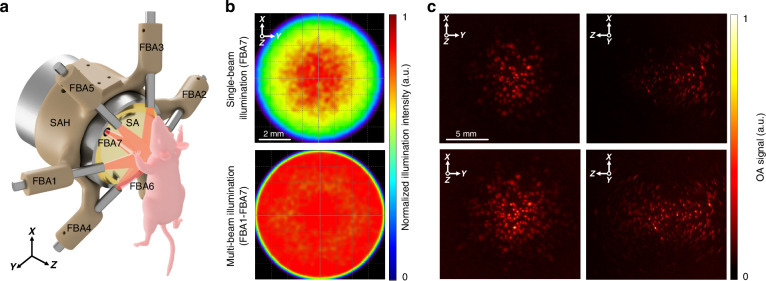
**The volumetric optoacoustic tomography (VOT) system employing hexagonally-shaped light delivery for noninvasive whole-heart imaging**. **a** Lay-out of the system assembly comprising a spherical array (SA), a 3D printed spherical array holder (SAH), fiber bundle arms (FBA1-FBA7), and the imaged animal. For uniform (multi-beam) illumination, all seven fiber bundle arms (FBA1-FBA7) were used. The FBA7, inserted in the central aperture of the array, was also used for single-beam illumination. **b** Comparison between single-beam (top row) and optimal multi-beam (bottom row) illumination profiles simulated with ray tracing. **c** Comparison of VOT reconstructions for the single-beam (top row) versus multi-beam (bottom row) illumination. Maximum intensity projection images along the *z*- (left column) and *y*- (right column) axis of the tissue-mimicking agar phantom embedded with a cloud of microspheres are shown

The experimental setup features a multi-beam illumination approach employing seven fiber bundle arms (FBAs, Fig. 1a). Ray tracing simulations have been performed to achieve a uniform light delivery over an 8-mm diameter circular region simulating the typical size of the mouse heart. Single-beam illumination with only one FBA (FBA7) inserted in the central aperture of the spherical transducer array only allowed for partial coverage of the imaged FOV (Fig. 1b, top). On the other hand, a multi-beam illumination approach employing all the seven FBAs (FBA1-FBA7) clearly enabled expanding the effective FOV of the spherical array to cover a volume matching that of the murine heart (Fig. 1b, bottom). The improved imaging performance of the multi-beam illumination approach was further confirmed in tissue-mimicking phantom experiments (Fig. 1c) where the VOT datasets were collected using either all seven FBAs or only FBA7, i.e., the arm that was inserted through the central aperture of the spherical array transducer. Specifically, the multi-beam illumination scheme results in significantly larger number of visible microspheres (~379) compared to single-beam illumination (~210) in the reconstructed images. Microspheres at the periphery of the phantom and at deep locations could only be rendered when all FBAs were used for delivering light into the phantom. The maximum depth achieved with all FBAs is ~14.45 mm compared that of 1 FBA (~9.5 mm). Note that, in both cases, the limited-view effects and directivity of the array’s elements still lead to spatial resolution degradation at peripheral positions away from FOV’s center^17^.

**Fig. 2:**
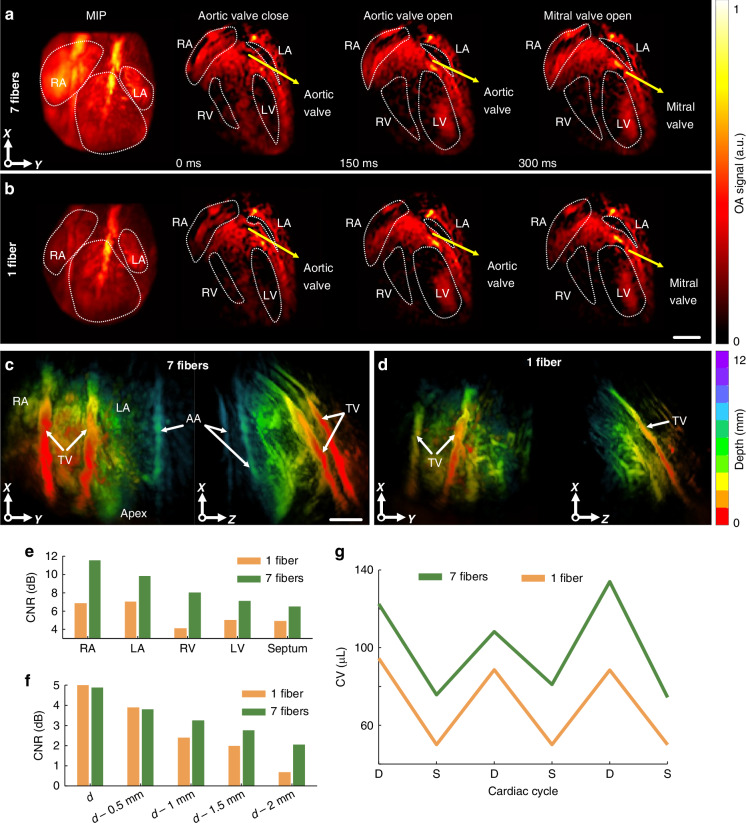
**In-vivo cardiac VOT imaging of the murine heart**. **a** Maximum intensity projections (MIPs) and cross-sections along the *z* axis of the 3D images obtained with multi-beam illumination at three different time points. Opening and closing of the aortic and mitral valves are indicated. LA - left atrium, LV - left ventricle, RA - right atrium, RV - right ventricle. **b** Equivalent images for single-beam illumination (Scale bar: 3 mm). **c** Color-coded MIP images along the *z* and *y* axis obtained with multi-beam illumination. TV - thoracic vessels; AA - abdominal aorta (Scale bar: 3 mm). **d** Equivalent images for single-beam illumination. **e** Contrast-to-noise ratio (CNR) at different anatomical regions in the heart for the images obtained with single-beam and multi-beam illumination. **f** CNR at increasing imaging depths (d to d–2 mm) for images acquired with single-beam and multi-beam illuminations. **g** LV measurements at different phases of the cardiac cycle. Measured end systole volume (ESV) and end diastole volume (EDV) are shown for single-beam and multi-beam illumination schemes. S Systole, D Diastole

Performance of the newly developed system was subsequently tested for in vivo imaging of the murine heart. The enhanced FOV achieved with the multi-beam scheme becomes evident in the maximal intensity projections (MIPs) and anatomical cross-sections along the depth axis (Fig. 2a, b). Clear delineation of the mouse heart along its long and short axes is achieved with the multi-beam illumination scheme, with the heart apex also clearly identified. The higher signal intensity observed in the thoracic vessels (TV) is mainly attributed to the broader light fluence distribution on the chest surface. The more uniform profile of light diffusion across the heart volume results in an expanded FOV at deeper regions. Larger areas of key anatomical structures, including the left ventricle (LV), the right ventricle (RV), the left atrium (LA), the right atrium (RA), and the apex are covered with multi-beam illumination. In addition, the systolic (ventricular contraction) and diastolic (ventricular filling) phases of the cardiac cycle can be better differentiated by looking at the states of the aortic and mitral valves, which are not clearly visible with the single-beam illumination. The mitral valve was open during diastole, allowing for LV relaxation and filling. Conversely, the aortic valve was open during systole, allowing for LV contraction to pump blood to the body through the aorta.

The increased effective penetration depth achieved with multi-beam compared to single-beam illumination was further verified in the lateral projections of the volumetric images (Fig. 2c, d). Here, it was possible to visualize deep structures such as the abdominal aorta (AA) up to ~10 mm depth (Fig. 2c, right), whereas an insufficient imaging depth of <7 mm was achieved with the single-beam illumination (Fig. 2d, right). Image quality was further quantified by calculating contrast-to-noise ratio (CNR) values for different anatomical regions of the mouse heart. Overall, CNR values were higher for multi-beam versus single-beam illumination for all selected heart regions (Fig. 2e, RA, LA, RV, LV, septum). More specifically, the calculated CNR values were RA: 6.81–11.54, LA: 7.02–9.83, RV: 4.08–7.98, LV: 4.98–7.08, septum: 4.87–6.46 for single- and multi-beam illumination schemes, respectively. Beat-by-beat analysis of cardiac dynamics was performed with 4D (time-lapse) VOT data.

Dependence of image quality on depth was further assessed by calculating the CNR metrics for both single-beam and multi-beam illumination patterns (Fig. 2f). The CNR was calculated as the difference between the mean signal intensity within the region marked in Fig. 2a (green rectangle) and 2B (orange rectangle) and the mean background intensity, normalized by the standard deviation of the background. As expected, the CNR decreased due to signal attenuation with depth. Yet the multi-beam configuration consistently yielded higher CNR values with increasing imaging depth while manifesting superior contrast uniformity across the entire imaged volume.

The comprehensive 4D data can potentially be used to obtain other functional information, e.g., to detect abnormal myocardial contraction, ventricular remodeling, or heart failure. Indeed, myocardial infarction following occlusion of coronary arteries leads to cardiac hypertrophy, adversely altering the ventricular anatomy and function^20,21^. Accurate quantification of cardiac volumes is of importance for the diagnosis of contractile abnormalities associated with alterations in cardiac output. In our measurements, the measured end systolic volume (ESV) of the LV was 20.6 ± 2.1 μL and 13.2 ± 2.0 μL with multi-beam and single-beam illumination schemes, respectively (Fig. 2g). The corresponding measured values of the end diastolic volume (EDV) were 48.1 ± 3.6 μL and 28.6 ± 2.0 μL, respectively (Fig. 2g). This underestimated values of the LV volume for single-beam illumination (7.4 μL lower during ESV and 19.5 μL lower during EDV) can result in substantial inaccuracies in the assessment of cardiac output.

**Fig. 3:**
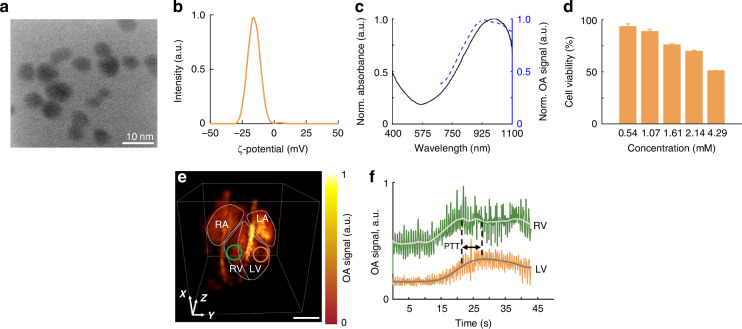
**Contrast-enhanced cardiac VOT with PEG-CuS nanoparticles**. **a** Transmission electron microscopy (TEM) image of PEG-CuS nanoparticles. **b** Zeta potential of PEG-CuS nanoparticles. **c** UV-Vis-NIR extinction and optoacoustic absorption spectra of PEG-CuS nanoparticles. **d** Cell toxicity of the nanoparticles at different concentrations performed on Chinese Hamster Ovarian (CHO) cell culture using an alamarBlue assay. **e** VOT image of the mouse heart before the start of intravenous injection of PEG-modified CuS nanoparticles (40 mM, 80 μL) (Scale bar: 3 mm). LA left atrium, LV - left ventricle, RA - right atrium, RV - right ventricle. **f** OA signal change observed within the defined regions in RV and LV marked by green and orange circles in **e**, respectively. The pulmonary transit time (PTT), estimated as the time delay between the PEG-CuS bolus appearance in RV and LV, is indicated. Scale bar: 3 mm

Importantly, VOT imaging based on endogenous contrast mainly relies on light absorption by hemoglobin in blood. This facilitates angiographic imaging but, at the same time, reduces the achievable penetration, thus hampering the assessment of blood flow dynamics in vital organs, particularly in the blood-filled heart chambers. Contrast-enhanced VOT imaging could then improve visibility of cardiac structures. Nanoparticles with absorption spectra extending into the NIR-II spectrum are of particular importance, given the reduced absorption and scattering of biological tissues in this wavelength range^22^. Herein, we explored the performance of contrast-enhanced imaging of the murine heart with the newly developed system assisted with polyethylene glycol (PEG)-coated copper sulfide (CuS) nanoparticles^23^. The nanoparticle synthesis was first validated by measuring the diameter distribution (9.75 ± 4.48 nm) in transmission electron microscopy (TEM) images (Fig. 3a). The measured zeta potential of a suspension of nanoparticles (−15.6 ± 0.98 mV) was further attributed to the attachment of long chains of PEG molecules (Fig. 3b). The synthesized PEG-CuS nanoparticles exhibited strong optical absorbance extending well into the NIR-II range (solid black line in Fig. 3c), which was confirmed with the optoacoustic measurements (dashed blue line in Fig. 3c). Thus, enabling contrast-enhanced imaging at 1064 nm excitation. Cell viability following exposure to a suspension of nanoparticles remained high for a broad concentration range (Fig. 3d). Specifically, the alamarBlue signal in Chinese Hamster Ovarian (CHO) cultured cells incubated with 0.54, 1.07, 1.61, 2.14, 4.29 mM suspensions of PEG-CuS particles was 93.1, 88.7, 75.7, 69.6, 50.9% with respect to the control group (CHO cells incubated with phosphate-buffered saline (PBS)), respectively. Real-time VOT imaging of the mouse heart during intravenous injection of PEG-CuS nanoparticles suspended in PBS resulted in improved cardiac contrast (Fig. 3e). The high temporal resolution of VOT further enabled the assessment of cardiovascular perfusion dynamics and quantification of the pulmonary transit time (PTT). PTT values of 1.765 ± 0.195 s were measured as the time delays between the PEG-CuS bolus appearance, as recognized by the VOT signal enhancement at selected voxels in the chambers (RV and LV) of the heart following injection of the nanoparticles (Fig. 3f).

## Discussion

Optoacoustic imaging has recently enabled high-frame-rate four-dimensional visualization of murine cardiovascular dynamics without the need for cardiac gating^10^. Cardiac dynamics has been assessed in murine hearts under hypoxic conditions^12^, and in myocardial infarct models^13^ at high temporal (100 Hz) and spatial (200 μm) resolution. The VOT imaging system presented in this study, combined with contrast-enhanced imaging in the NIR-II window, marks a significant advancement in the investigation of cardiac morphology and fast biodynamics in small animal models. The multi-beam illumination scheme, designed to uniformly illuminate the chest, played a critical role in expanding the effective FOV and achieving sufficient penetration depth to cover the entire volume of the murine heart. The VOT images acquired from a tissue-mimicking phantom and a murine heart in vivo corroborated the enhanced performance with respect to the previously reported implementations. In particular, accurate delineation of internal cardiac structures, improved characterization of systole and diastole phases of the cardiac cycle (ventricular filling and contraction), as well as increased visibility of the mitral and aortic valve dynamics, could be achieved. Also important is the fact that deep-seated structures such as the AA became visible with an increased ~10 mm imaging depth achieved with the proposed system. Presently, the limited depth achieved with optoacoustic imaging systems represents a major hinderance for in vivo imaging of the mouse heart and other organs. While visibility of deep-seated structures can potentially be improved with algorithms accounting for light and/or ultrasound attenuation, such corrections generally do not result in enhanced contrast-to-noise (CNR) performance as the collected noise is equally amplified with depth. Our results indicate that an improved CNR was achieved for all selected heart regions, practically resulting in more accurate readings of indicators of cardiac dynamics and function, such as LV volumes, ESV, and EDV. All those are important prognostic indicators for many cardiac diseases, such as mitral valve regurgitation, heart failure, and LV remodeling^24^. Hence, underestimation of LV volume with single-beam illumination could potentially result in poor assessment of these diseases.

Pulse-echo US imaging has been an indispensable tool for revealing morphological and functional information relevant for the characterization of cardiovascular diseases through the analysis of the size, mechanical properties, and motion of the heart walls or other structures^25^. Doppler US or contrast-enhanced ultrasound (CEUS) imaging has further been shown to enable visualization of blood flow, ischemia, and angiogenesis^26,27^. Yet, US imaging relies on line-by-line acquisition or compounding of multiple frames to achieve sufficient CNR, which practically limits the 3D dynamic imaging performance. The system introduced in this work can capture a volumetric image of the entire heart with a single-shot (one laser pulse) excitation. The 4D imaging at 200 volumes per second, as achieved in this work, is generally sufficient to accurately capture the fast murine cardiac motion on a beat-by-beat basis and the dynamics of internal structures such as the cardiac valves^3,12^.

The volumetric frame rate of the developed VOT system (200 Hz) was dictated by the pulse repetition rate of the laser. With this high frame rate, we were able to visualize the cardiac dynamics on a beat-to-beat basis without the need for signal averaging, thus averting image artifacts related to inter-frame heart motion. Note that the optoacoustic signals have been generated almost instantaneously (within a <10 ns duration of the laser pulse) across the entire heart. For such short duration, the heart motion is negligible, and thus no blurring occurs in the images acquired with a single laser pulse. During analysis, we discarded frames in which breathing motion was observed by employing self-gated respiratory motion rejection algorithm^28^. While the 200 Hz frame rate was sufficient to accurately capture the heart perfusion parameters in 3D, it has been shown that significantly faster VOT at kilohertz frame rates is possible when employing compressed sensing signal acquisitions^29^, which further enabled visualizing cardiac mechanical waves in murine hearts^30^. Such imaging speed may outperform US imaging for studying heart diseases in preclinical mouse models. Note, however, that VOT imaging in larger animals and humans is still severely hampered by the strong attenuation of light relative to US waves.

VOT has previously been employed for assessing cardiac functional parameters such as PTT^10,12,13^, yet the measurements were hampered by the shallow penetration depth of several millimeters. In this work, we achieved whole-heart imaging coverage by using the PEG-CuS nanoparticles, which exhibited a favorable toxicity profile and strong absorption at 1064 nm. This is a particularly important wavelength considering the relatively low absorption of hemoglobin and water, the reduced scattering of light, as well as broad availability of Q-switched Nd:YAG lasers providing high per-pulse energy and pulse repetition frequencies at this wavelength. Perfusion of the mouse heart with PEG-CuS nanoparticles also resulted in a clearly enhanced contrast of the heart chambers and other vascular structures, facilitating e.g., visualization of perfusion in coronary arteries. The PTT is an important biomarker of heart disease that has previously been shown to play an important role in assessing heart function following myocardial infarction or hypoxic stress^13,31^. VOT imaging at 1064 nm assisted with PEG-CuS particles can thus facilitate quantification of the PTT under these conditions where bleedings or scars often obscure visibility of the heart chambers at shorter wavelengths. The current experiments were mainly aimed at demonstrating the basic capacity of the developed VOT system to assess rapid cardiac dynamics at the whole heart scale, with only terminal experiments performed under the current conditions. Long-term toxicity effects of the CuS nanoparticles should be further assessed in order to enable longer duration studies.

While VOT has demonstrated strong capabilities in imaging the murine heart, its application to larger animals or humans remains constrained by major technical challenges, including limited light penetration and acoustic distortions due to lungs and ribs in the thoracic region. The high-resolution, real-time volumetric imaging achieved in mice relies on their relatively small heart size and short light penetration paths, which do not directly translate to larger species. The clinical translation of VOT for cardiac imaging in humans is challenging, considering the depth of the heart and the presence of thick bones in the chest. Nonetheless, several strategies could be explored to alleviate these challenges, such as utilizing broader light illumination patterns with increased per-pulse energy, deploying larger transducer arrays with lower central frequency, wider acceptance angles, and enhanced sensitivity, as well as improving reconstruction algorithms.

Overall, this work demonstrates the importance of developing optimized illumination schemes for pushing the performance envelope of VOT. The proposed imaging system achieved imaging performance superior to the currently available solutions by operating at fast imaging rates of 200 volumes per second and employing a multi-beam illumination scheme for uniform light delivery to the target area. Contrast-enhanced OA imaging with PEG-CuS nanoparticles featuring strong absorption within the NIR-II spectral window further facilitated visualization and quantification of perfusion dynamics. These unique capabilities are expected to facilitate monitoring of heart diseases and the assessment of therapeutic interventions, thus driving substantial progress in preclinical cardiovascular research.

## Materials and methods

### Cardiac volumetric optoacoustic tomography system

The custom-designed VOT imaging platform comprises light delivery elements, stages for mechanical scanning, a spherical US transducer array, and a data acquisition system (DAQ). Optical excitation was achieved using an optical parametric oscillator (OPO) laser (Innolas GmbH, Krailling, Germany) that provides <10 ns duration pulses at a repetition rate of 200 Hz over a broad tunable wavelength range from 660 up to 1300 nm. In this work, 1064 nm excitation wavelength was used in all experiments to facilitate deeper tissue penetration. The per-pulse energy of the output light beam at this wavelength was ~100 mJ. The beam was coupled to a custom-made fiber bundle (Nanjing Technology LTD, Jiangsu, China) that bifurcates into seven individual outputs delivering in total 35 mJ per-pulse output energy (35% coupling efficiency, 5 mJ per-pulse for each output). One of the seven fiber bundle arms was inserted into the central cavity of the spherical array, and the remaining six outputs were equally spaced at 40 mm distance from the center of the array and directed towards the imaged FOV (Fig. 1a). The specific positions and orientations of the arms were established based on ray-tracing simulations, as described below. The optical fluence was maintained below exposure limits established by the laser safety standards for skin exposure, i.e., ~100 mJ/cm² at 1064 nm^32^.

The spherical US array used for collecting generated optoacoustic signals consisted of 512 piezocomposite elements uniformly distributed across a spherical surface with 40 mm radius and 110° (0.85*π* solid angle) angular coverage (Imasonic SAS, Voray, France). The individual piezoelectric elements have a central detection frequency of 7 MHz and ~85% (full width at half maximum) detection bandwidth. The size, distribution, and frequency response of the elements determine an effective FOV of approximately 1 cm^3^ around the spherical center, suitable for volumetric imaging of the entire mouse heart.

The optoacoustic responses were captured and digitized by a custom-made DAQ (Falkenstein Mikrosysteme GmbH, Taufkirchen, Germany) operating at 40 megasamples per second. Synchronization with the laser pulses was achieved by triggering the DAQ with the Q-switch output of the OPO laser. Simultaneously, 512 time-resolved signals (256 samples each) corresponding to all elements of the array were acquired for each laser pulse. Overall, the VOT data acquisition time was set to 3 min, corresponding to 36,000 frames at a PRF of 200 Hz. The digitized signals were then transferred to a personal computer (PC) via Gb/s Ethernet connection, where they were stored for subsequent image reconstruction and analysis.

### Phantom preparation

The illumination uniformity and imaging depth were validated by imaging a cloud of randomly distributed 50 µm diameter light-absorbing polyethylene microspheres (Cospheric LLC, Goleta, United States) embedded in a tissue-mimicking agar phantom. The phantom was prepared by first dissolving an agar powder (1.3% by weight) in distilled water (DI). The warm agar-water mixture was then mixed with Indian ink and Intralipid (6% by volume) to achieve an absorption coefficient of ${{\mu }_{a}=0.23{cm}}^{-1}$ and a reduced scattering coefficient of ${{\mu ^{\prime} }_{s}=10{cm}}^{-1}$. The polyethylene microspheres were mixed with 4 mg/ml tannic acid aqueous solution and vigorously stirred for 1 min using a Vortex Genie 2 shaker. After that, the microsphere suspension was placed under a tip sonicator for 3 min to ensure uniform dispersion. The spheres were then added to the tissue-mimicking liquid and left at 4 °C for rapid solidification.

### Ray-tracing simulations

Ray tracing simulations were performed to determine the optimal arrangement of the fiber bundle arms around the spherical transducer array. Specifically, simulations were performed in TracePro (Lambda Research Corporation) to uniformly illuminate a 8 mm diameter sphere located at the center of the array. Important factors such as the fiber bundle arm diameter (4.5 mm), half angle of the output beam (11.04°), numerical aperture (0.28), transducer array diameter (80 mm), focal distance (40 mm), and diameter of the central cavity (8 mm) of the array were considered. The estimated angles (in degrees) with respect to the [X Y Z] axes for each fiber bundle arm (FBA, Fig. 1a) providing the most homogeneous illumination were $\pm [6600]$ for FBA1 and FBA2, $\pm [27600]$ for FBA3 and FBA4, $\pm [27\,-600$] for FBA5 and FBA6, and [0 0 0] (central aperture of the array) for FBA7, respectively.

### Synthesis and characterization of PEG-coated CuS nanoparticles

The polyethylene glycol (PEG)-coated copper sulfide (CuS) nanoparticles used as contrast agent for cardiac imaging at 1064 nm excitation were synthesized with a protocol adapted from elsewhere^23^. First, citrate-capped CuS nanoparticles were prepared by mixing CuCl_2_ (50 mM, 1 mL) solution and sodium citrate tribasic dihydrate (34 mM, 1 mL) solution in 48 mL of deionized (DI) water in a round-bottom flask under stirring at room temperature, followed by addition of Na_2_S (1 M, 100 μL) for 5 min. PEGylation was done to increase the circulation time of the CuS nanoparticles for better visualization of the cardiac perfusion and estimation of PTT. The resulting dark-brown liquid was placed in an oil bath at 90 °C for 15 min until its color turned dark green. An ice-water bath was employed to achieve rapid cooling of the flask. Centrifugal filter units were used to remove the unreacted components from the resulting solution at 3350 RCF for 15 min using a Multifuge 1S-R (Thermo Scientific, Germany). A total of 12 mg of MeO-PEG-SH was dissolved in 5 mL of DI water and mixed with above citrate-capped CuS nanoparticles overnight for ligand exchange. Finally, the synthesized PEG-CuS nanoparticles were concentrated to 1 mL of DI water and stored at +4 °C.

The PEG-CuS morphology was analyzed using transmission electron microscopy (TEM). The particles were drop-casted on a holey carbon film (Quantifoil R 2/1) supported on a Cu 300- mesh grid with a 2 nm of carbon film, and dried with filtering paper. The remaining water was evaporated at room temperature for 1 h. A JEOL JEM-F200 HR-TEM system was operated at 200 kV accelerating voltage and 20 µA electron gun emission using a Gatan Rio 16 camera at different magnification ratios.

The zeta potential of the nanoparticles was measured using a Zetasizer Nano ZS in DTS1070 folded capillary zeta cell after 10-fold dilution. The absorption spectrum for the wavelength range 420–1100 nm was collected using a quartz glass cuvette (CV10Q7F, Thorlabs) in a UV-1900i spectrophotometer (Shimadzu, Japan).

The biocompatibility of CuS-PEG nanoparticles was assessed using a standard alamarBlue cell metabolism assay on a Chinese Hamster Ovarian (CHO) cell culture after 24 h co-incubation. Specifically, the fluorescence from the reduced dye at 540 nm excitation and 590 nm emission wavelengths was measured. Data was normalized to the signal from phosphate-buffered saline (PBS)-fed cells.

### Animal preparation and in vivo VOT imaging experiments

Female Hsd:AthymicNude Foxn1nu/nu mice (6–8 weeks old) were employed for in vivo imaging in accordance with the regulations outlined in the Swiss Federal Act on Animal Protection. The experimental procedures were approved by the Cantonal Veterinary Office Zürich. The mice were kept in ventilated cages in a room where temperature (21 ± 1 °C) and humidity (55 ± 10%) were controlled, with a 12-h reversed light/dark cycle. They had unrestricted access to pelleted food and water. During the imaging sessions, the mice were positioned vertically inside a water tank using a custom-made animal holder^19^. The mice remained under isoflurane anesthesia (Abbott, Cham, Switzerland, 5% volume ratio for induction and 1.5% volume ratio for maintenance in an oxygen/air mixture with flow rate 100/400 mL/min) with fore and hind paws secured to a specifically designed holder. Gas anesthesia was delivered via a custom-made breathing mask attached to a mouth clamp, ensuring that the animal’s nose and mouth were always positioned above the water surface. The position of the spherical array together with all fiber bundle arms was adjusted with motorized translational and rotational stages for optimal visualization of the heart. The water temperature was maintained at 36 °C throughout the experiments using a feedback-controlled heating stick. To prevent dehydration and provide protection from laser light, a vet ointment (Bepanthen, Bayer AG, Leverkusen, Germany) was applied to the eyes of the animal. During each VOT imaging session, 60 s of baseline data were acquired followed by intravenous injection of a bolus of 80 $\mu L$ PEG-coated CuS-based microparticles with a concentration of 40 mM. Note that the contrast agent is further diluted in blood by ~10X when injected intravenously. Images were acquired during (~15 s) and after (~105 s) injection of the particles. The animals were kept alive for the entire duration of the experiment and then sacrificed without waking up.

### VOT image processing and reconstruction

The acquired raw signals were deconvolved with the impulse response of the transducer array elements and band-pass filtered between 0.1 and 8 MHz to suppress the low-frequency noise^33^. The transducer elements were split into 9 equally spaced virtual sub-elements, i.e., tomographic up-sampling, to reduce the streak-type artifacts and thus to increase the image contrast^17^. Individual volumetric images for each acquired frame were reconstructed with voxel size of 0.05 × 0.05 × 0.05 mm^3^. Volumetric image reconstruction was sequentially performed using a 3D back-projection algorithm implemented on the graphics processing unit (GPU)^34^. Note that all the images were reconstructed using manually optimized average speed of sound to maximize the sharpness and contrast of certain heart features^35^. Furthermore, the negative values in the reconstructed images, arising from the inherent inaccuracy of the back-projection algorithm, were set to zero, as they do not contribute to the initial pressure rise. Self-gated motion rejection algorithm was employed to discard the frames in which breathing motion was observed^28^. For six time points, left ventricle (LV) areas were determined through manual segmentation, and the LV end diastolic volume (EDV) and end systolic volume (ESV) in microliters ($\mu L$) were calculated by multiplying the LV area (${{mm}}^{2}$) by the LV length (${mm}$).

## Data Availability

The data is available from corresponding author upon reasonable request.
